# Identification and validation of a major QTL on chromosome 2A for wheat-*Parastagonospora nodorum* interactions

**DOI:** 10.1186/s42483-025-00371-z

**Published:** 2025-09-24

**Authors:** Cong Li, Xinyao He, Jian Ma, Pawan K. Singh

**Affiliations:** 1 International Maize and Wheat Improvement Center (CIMMYT), Texcoco, C.P. 56237 Mexico; 2 State Key Laboratory of Crop Gene Exploration and Utilization in Southwest China, Triticeae Research Institute, Sichuan Agricultural University, Chengdu, 611130 China; 3 Crop Research Institute, Sichuan Academy of Agricultural Sciences, Chengdu, 610066 China

**Keywords:** Bread wheat, Septoria nodorum blotch, QTL mapping, Disease resistance

## Abstract

Wheat (*Triticum aestivum* L.) is frequently affected by Septoria nodorum blotch (SNB), a fungal disease that significantly reduces wheat yields. In this study, two recombinant inbred line (RIL) populations, developed from crosses involving two elite CIMMYT breeding lines (WUYA and KATH) and a common susceptible male parent (CIANO T79), were used to detect quantitative trait loci (QTL) associated with SNB resistance. High-density genetic maps were constructed for these RIL populations by incorporating presence/absence variation (PAV) markers using the DArTseq genotyping platform. Three major and stable QTL linked to SNB resistance were identified on chromosomes 2A, 4B, and 5B. Among these, *QSnb.cim-2A* accounted for 22.16%–28.74% and 17.62%–19.71% of the phenotypic variation in the WUYA/CIANO T79 and KATH/CIANO T79 populations, respectively, and it was also validated in the CASCABEL/CIANO T79 RIL population. The remaining two QTL, *QSnb.cim-4B* and *QSnb.cim-5B*, were found to be associated with *Rht-B1b* and *tsn1*, respectively. The combined effect of these three QTL significantly improved SNB resistance while also reducing plant height, indicating their promising utilization in wheat breeding programs.

**Supplementary Information:**

The online version contains supplementary material available at 10.1186/s42483-025-00371-z.

## Background

Wheat (*Triticum aestivum* L.) is one of the most important cereal crops in the world and plays a crucial role in human food security (Acevedo et al. 2018). However, wheat productivity is continuously threatened by biotic stress (Figueroa et al. 2018). Septoria nodorum blotch (SNB), caused by a filamentous ascomycete fungus *Parastagonospora nodorum*, leads to wheat leaf and glume blotch and is a devastating fungal pathogen of both common and durum wheat (Downie et al. 2021; Peters Haugrud et al. 2022). Disease epidemics have been reported in nearly all wheat-producing regions with warm and humid growing conditions, particularly the USA, Australia, parts of Europe, and southern Brazil (He et al. 2022). This disease can decrease wheat quality and cause significant yield losses by up to 50% under conducive conditions (Eyal 1987). Because the pathogen has a short incubation period and secretes necrotrophic effectors that accelerate infection, fungicide applications are heavily relied upon in the field (He et al. 2022; Lin et al. 2022). As a sustainable and environmentally friendly approach, resistance breeding is often preferred for managing SNB.

Wheat and *P. nodorum* interact in an inverse gene-for-gene manner, with host sensitivity genes recognizing *P. nodorum* necrotrophic effectors (NEs) (Faris and Friesen 2020). When the host recognizes the SNB pathogen, a defense response is activated, resulting in programmed cell death of the surrounding leaf tissue (Downie et al. 2021). This response aims to limit the pathogen’s spread (McDonald 2025). However, as a necrotroph, *P. nodorum* exploits the dying tissue as a nutrient source, facilitating disease progression (Oliver et al. 2012). Currently, based on the above interaction model, a total of nine sensitivity gene-NE interactions have been characterized, including *Tsn1*-SnToxA, *Snn1*-SnTox1, *Snn2*-SnTox267, *Snn3-B1*-SnTox3, *Snn3-D1*-SnTox3, *Snn4*-SnTox4, *Snn5*-SnTox5, *Snn6*-SnTox267, and *Snn7*-SnTox267 (Peters Haugrud et al. 2022). So far, only three sensitivity genes in wheat have been cloned, including *Tsn1* (located on chromosome 5BL), *Snn1* (1BS), and *Snn3-D1* (5DS) (Faris et al. 2010; Peters Haugrud et al. 2022). Additionally, many quantitative trait loci (QTL) have been identified using bi-parental mapping and genome-wide association study (GWAS) methods. For example, *QSnb.niab-2A.3* identified on chromosome 2A had significant effects on field resistance to both leaf blotch and glume blotch (Lin et al. 2020). *QSnb.cim-5B.2* with 19.51% phenotypic variation explained (PVE) was very close to *Tsn1*, and the QTL also conferred resistance to tan spot (Singh et al. 2019). Hu et al. (2019) also identified the same QTL in their study that was associated with both tan spot and SNB resistance. In a recent GWAS study on CIMMYT and South Asian germplasm, marker-trait associations (MTAs) were identified on chromosomes 5B, 5A, and 3D, with those surrounding *Tsn1* being the most significant (Mandal et al. 2024). In another study, 50 MTAs associated with SNB resistance were detected, and six of them on chromosomes 2A, 2B, 3B, 5B, and 7A were considered robust and stable (Lin et al. 2022).

Mining and utilizing genes/QTL associated with SNB resistance are essential for wheat breeding programs focused on disease resistance. Therefore, ongoing efforts focus on screening, characterizing, verifying, and transferring stable resistance genes from diverse sources, as well as eliminating susceptibility factors, to improve both the level and diversity of resistance (Singh et al. 2019). In our previous studies, two CIMMYT-resistant lines, WUYA and KATH, have exhibited excellent resistance to spot blotch, and they were used to develop two recombinant inbred line (RIL) populations to dissect the underlying genetic factors (He et al. 2020; Singh et al. 2018). In this study, the two populations were evaluated for SNB resistance in the greenhouse to identify consistent QTL and their flanking markers, and a major QTL was validated in an additional population for its robustness and phenotypic effects.

## Results

### Phenotypic analysis

**Fig. 1:**
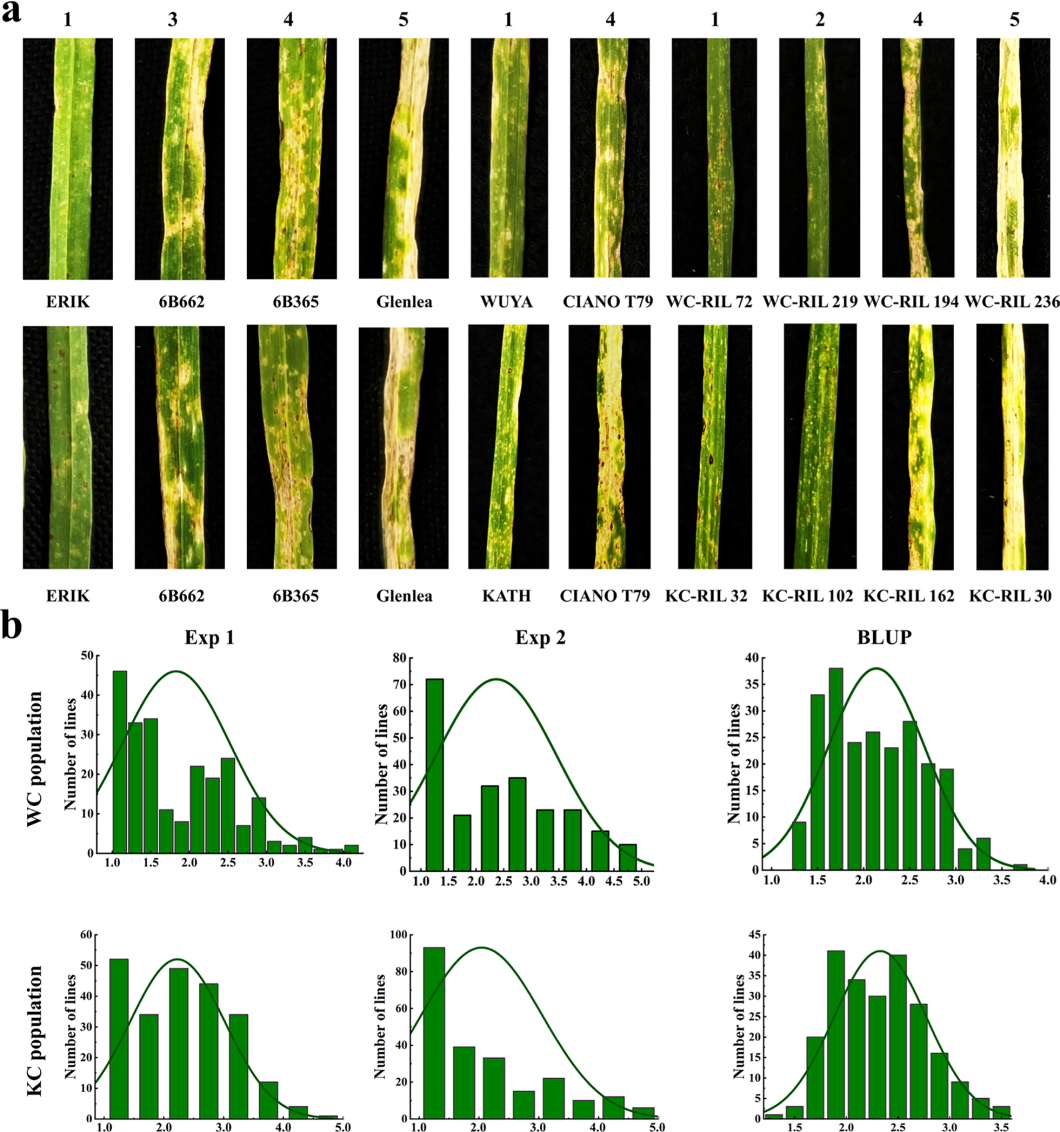
Phenotypic variation of the parents and RILs in the WC and KC populations. **a** Septoria nodorum blotch (SNB) symptoms of the parents WUYA, KATH**,** CIANO T79, and selected lines with different levels of resistance in the WC and KC populations. The numbers 1 to 5 represent the different SNB reaction types. ERIK, 6B662, 6B365, and Glenlea were used as checks for SNB reaction types 1, 3, 4, and 5, respectively. **b** Histogram for SNB in different experiments. Exp, Experiments; BLUP, Best linear unbiased prediction

**Fig. 2:**
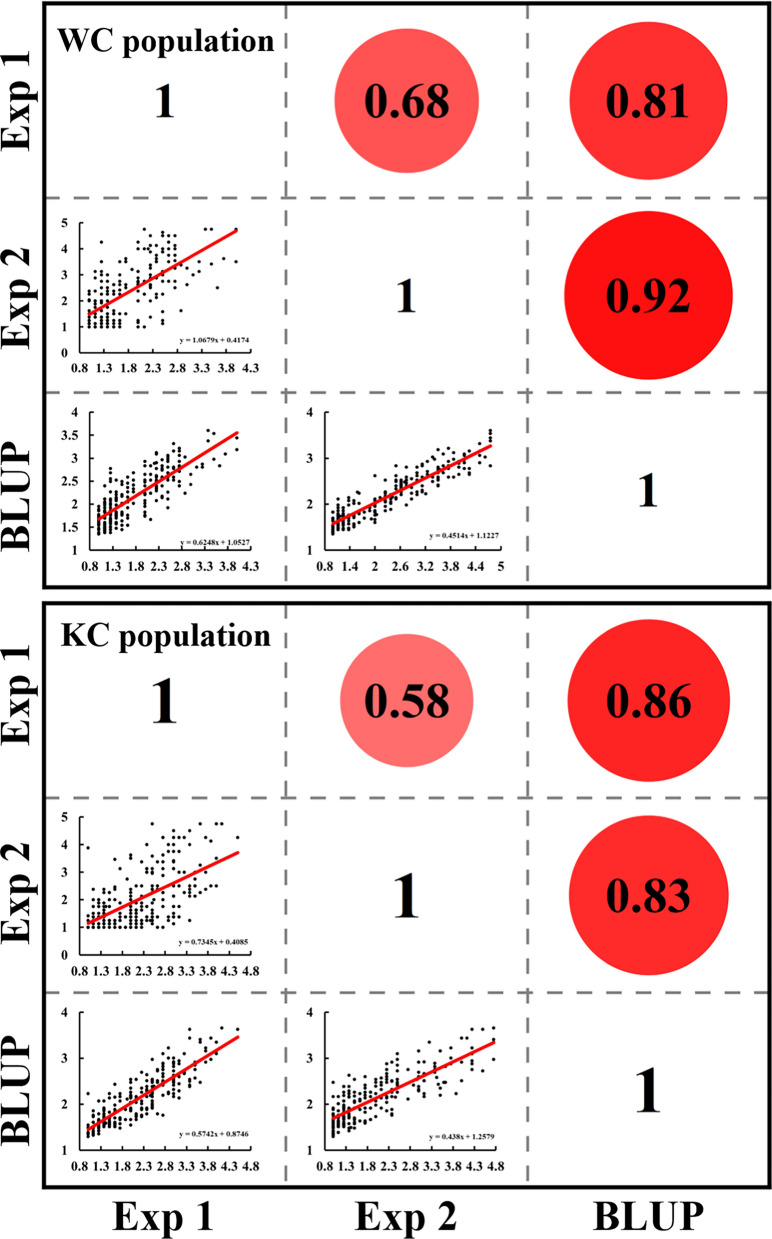
Correlation analysis for Septoria nodorum blotch (SNB) among different experiments and the BLUP dataset. Exp, Experiments; BLUP, Best linear unbiased prediction

A wide range of SNB resistance was observed in the two populations, and the female parents WUYA and KATH performed consistently better than the common male parent CIANO T79 (Fig. 1a). RIL lines of the WC and KC populations exhibited a continuous distribution in SNB resistance (Fig. 1b), and transgressive segregation was observed in both populations (Fig. 1a, Table 1). Both populations had lower standard deviations ranging from 0.62 to 1.08 in WC and from 0.53 to 1.00 in KC (Table 1). Correlation coefficients of SNB among the two experiments and BLUP values were high in both populations, ranging from 0.68 to 0.92 in the WC population and from 0.58 to 0.86 in the KC population (Fig. 2). ANOVA demonstrated the significant effects of ‘genotype’ and ‘genotype-by-experiment’ interaction (Table 2). Heritability estimates of 0.92 and 0.85 were obtained for the WC and KC populations, respectively, suggesting that SNB resistance was largely determined by genetic variation (Table 2).

**Table 1:** Basic statistics for the KC and WC populations

Population	Experiment	Parents		RILs			SD
		W/K	CIANO T79	Min	Max	Mean	
WC	Exp 1	1.13	1.63	1.00	4.00	1.81	0.69
	Exp 2	1.00	2.25	1.00	4.75	2.36	1.08
	BLUP	1.74	2.31	1.26	3.83	2.09	0.62
KC	Exp 1	1.13	3.00	1.00	4.50	2.24	0.79
	Exp 2	1.13	2.25	1.00	4.75	2.03	1.00
	BLUP	1.74	2.10	1.29	3.65	2.16	0.53

W, WUYA; K, KATH; C, CIANO T79; BLUP, Best linear unbiased prediction; Exp, Experiment; SD, Standard deviation

**Table 2:** Analysis of variance for SNB resistance in the WC and KC populations

Population	Source	DF	MS	*F*-value	*P*-value	*H*^2^
WC	Rep(Exp)	2	1.32	7.4	< 0.00	
	Genotype	230	2.62	14.72	< 0.00	
	Experiment	1	67.77	380.55	< 0.00	
	Genotype*experiment	230	0.63	3.55	< 0.00	
	Error	456	0.18			0.92
KC	Rep(Exp)	2	1.2	4.81	< 0.00	
	Genotype	230	2.47	9.84	< 0.00	
	Experiment	1	7.63	30.45	< 0.00	
	Genotype*experiment	230	0.65	2.59	< 0.00	
	Error	446	0.25			0.85

SNB: Septoria nodorum blotch; WC: The WUYA/CIANO T79 population; KC: The KATH/CIANO T79 population; *H*^2^: Broad-sense heritability; Exp: Experiment; DF: Degree of freedom; MS: Mean square

### QTL mapping

**Fig. 3:**
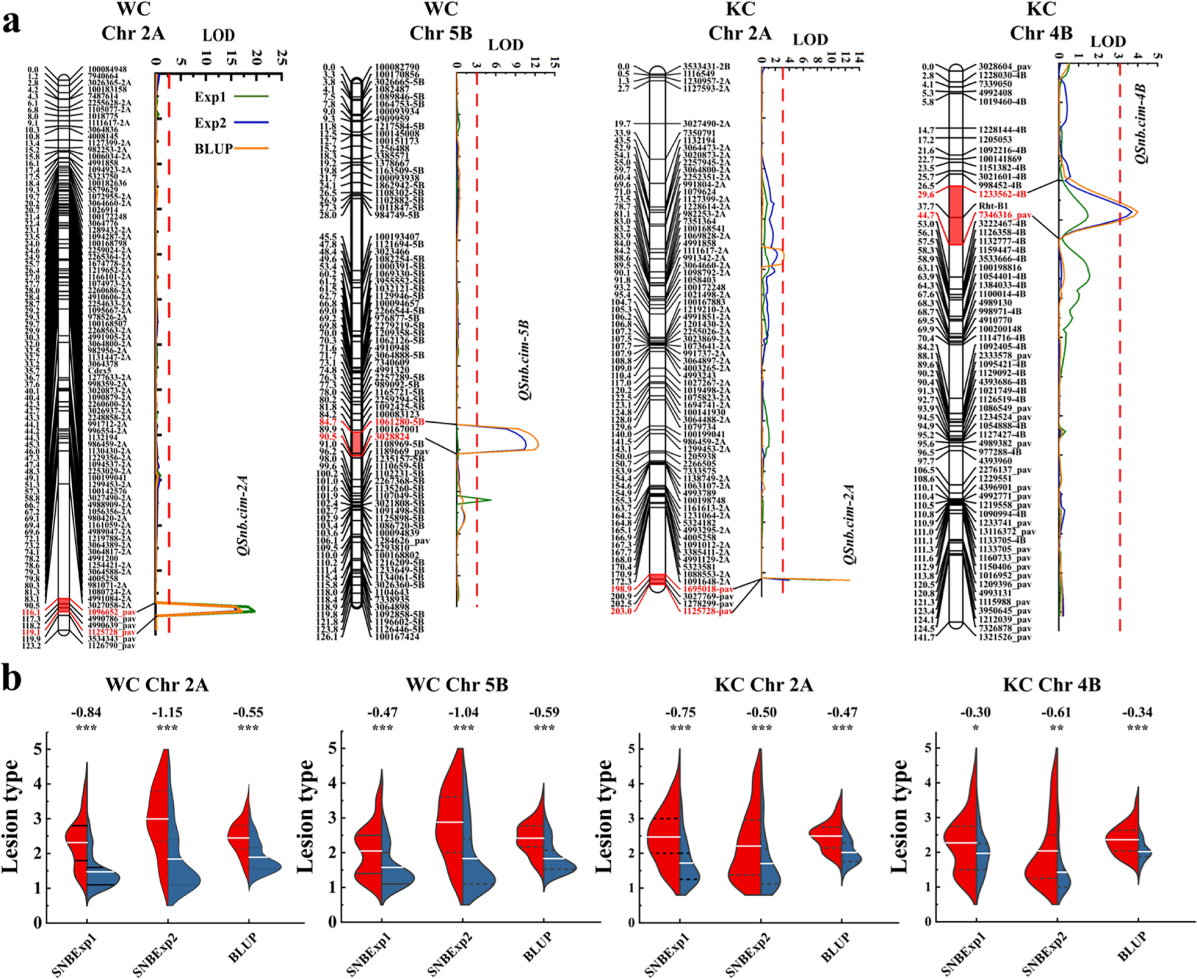
Genetic map and QTL profile for *QSnb.cim-2A* and *QSnb.cim-5B* in the WC population, and *QSnb.cim-2A* and *QSnb.cim-4B* in the KC population (**a**). Only framework markers are shown in the linkage maps. **b** Phenotypic differences between QTL groups are shown using violin plots, where blue and red represent RILs with and without the target QTL, respectively. *** and * indicate significance at *P* < 0.001 and *P* < 0.05, respectively. Differences between the compared groups are shown at the top of the violin plots

In the WC population, two major and stable QTL, *QSnb.cim-2Aw and QSnb.cim-5B* located on chromosomes 2A and 5B, respectively, were identified (Table 3, Fig. 3a). *QSnb.cim-2Aw* was detected in both experiments and the BLUP dataset, explaining phenotypic variation between 22.16% and 28.74%, whereas *QSnb.cim-5B* was only detected in one experiment and the BLUP dataset, explaining phenotypic variation between 15.25% and 21.79% (Table 3). These two QTL significantly reduced the mean SNB lesion type by 0.55 and 0.59, respectively (Fig. 3b).

**Table 3:** Major quantitative trait loci for SNB resistance identified in the WC and KC populations

Population	QTL	Chr	Exp	Position (cM)	Marker I	Marker II	LOD	PVE (%)	R source
WC	*QSnb.cim-2Aw*	2A	Exp1	119	*4990639_pav*	*1125728_pav*	19.60	28.74	W
		2A	Exp2	119	*4990639_pav*	*1125728_pav*	15.77	23.11	W
		2A	BLUP	119	*4990639_pav*	*1125728_pav*	16.79	22.16	W
	*QSnb.cim-5B*	5B	Exp2	89	*1061280-5B*	*100167001*	10.43	15.25	W
		5B	BLUP	89	*1061280-5B*	*100167001*	15.84	21.79	W
KC	*QSnb.cim-2Ak*	2A	Exp1	203	*1278299_pav*	*1125728_pav*	9.63	17.62	K
		2A	Exp2	202	*3027769_pav*	*1278299_pav*	12.33	19.71	K
		2A	BLUP	203	*1278299_pav*	*1125728_pav*	11.35	18.86	K
	*QSnb.cim-4B*	4B	Exp2	38	*Rht-B1*	*7346316_pav*	3.70	7.15	K
		4B	BLUP	38	*Rht-B1*	*7346316_pav*	4.00	6.66	K

SNB, Septoria nodorum blotch; WC, The WUYA/CIANO T79 population; KC, The KATH/CIANO T79 population; QTL, Quantitative trait loci; Chr, Chromosome; Exp, Experiment; BLUP, Best linear unbiased prediction; LOD, Logarithm of odds; PVE, Phenotypic variation explained; R source: W, WUYA; K, KATH

In the KC population, two stable QTL, *QSnb.cim-2Ak* and *QSnb.cim-4B*, were identified on chromosomes 2A and 4B, respectively (Table 3). The former had the same flanking marker (*1125728_pav*) as *QSnb.cim-2Aw*. It was detected in both experiments and the BLUP dataset, accounting for phenotypic variation between 17.62% and 19.71% (Table 3). This QTL can significantly reduce the mean SNB lesion type by 0.47 (Fig. 3b). *QSnb.cim-4B* was identified only in one experiment and the BLUP dataset, explaining phenotypic variation between 6.66% and 7.15% (Table 3). Despite the low phenotypic effects, *QSnb.cim-4B* was associated with the well-known dwarfing gene *Rht-B1b*, and reduced the SNB lesion type by 0.30 and 0.61 in the two experiments, respectively (Fig. 3b).

Three and seven major QTL were identified using multi-environment traits in the WC and KC populations, respectively (LOD = 5). *QSnb.cim-2Aw* and *QSnb.cim-5B* in population WC, and *QSnb.cim-2Ak* and *QSnb.cim-4B* in population KC were simultaneously detected by the multi-environmental algorithm, further suggesting that they were stable QTL (Additional file 1: Table S2). *QSnb.cim-2Aw* and *QSnb.cim-2Ak* had similar positions and shared the same flanking marker (*1125728-pav*), suggesting that they represent the same QTL on chromosome 2A, which was designated as *QSnb.cim-2A*.

### Effects of different allelic combinations in the WC and KC populations

**Fig. 4:**
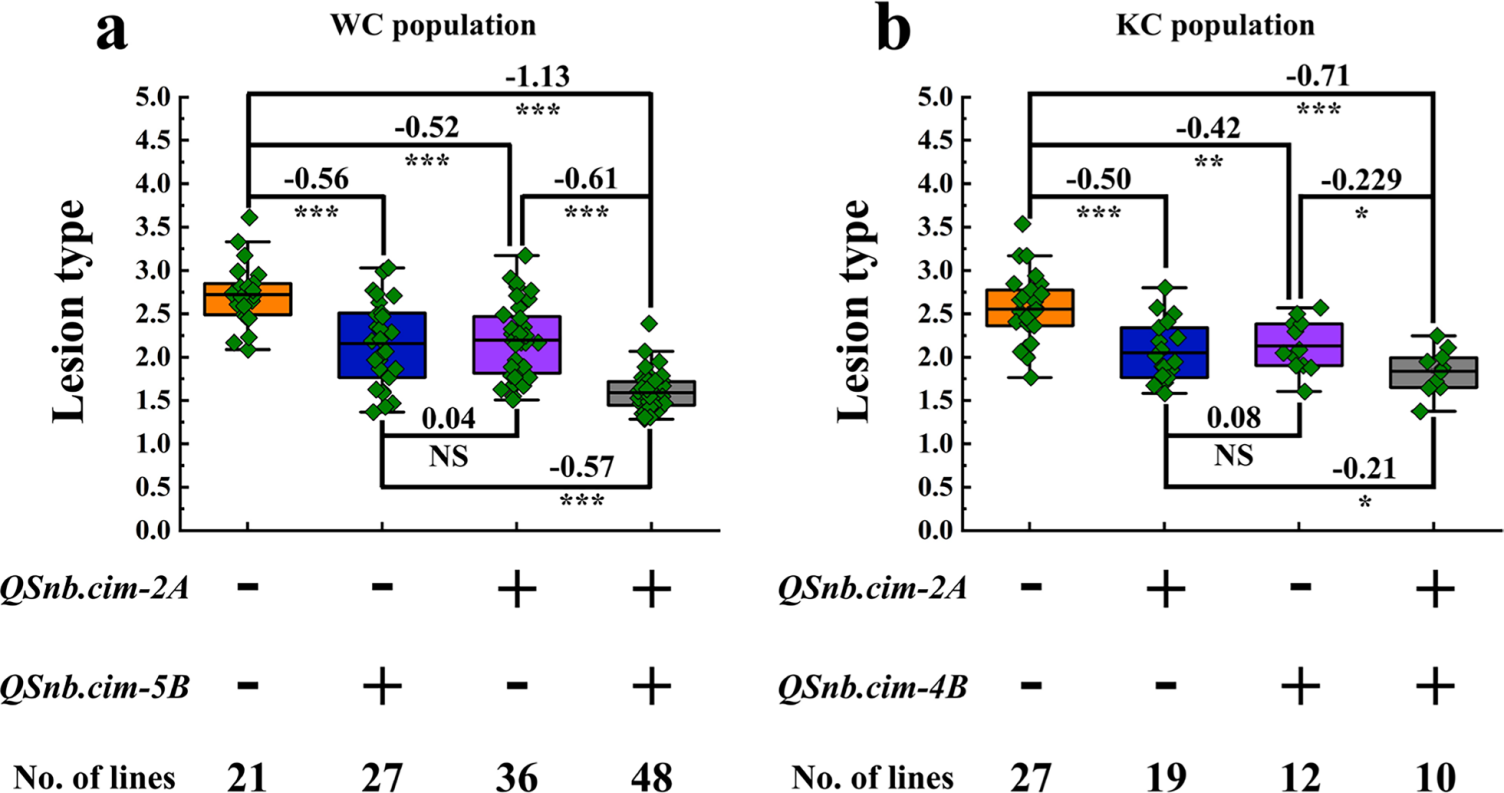
Phenotypic effects of *QSnb.cim-2A* and *QSnb.cim-5B* for Septoria nodorum blotch (SNB) in the WC population (**a**), and those of *QSnb.cim-2A* and *QSnb.cim-4B* in the KC population (**b**). + and − represent RILs with and without the target QTL, respectively. ***, ** and * represent significance at *P* < 0.001, *P* < 0.01, and *P* < 0.05, respectively. NS, No significant difference was identified. Differences between the compared groups are labeled at the top of the charts

In the WC population, compared with lines without any of the two QTL, those with one QTL exhibited a significant reduction in SNB. Lines with either *QSnb.cim-2A* or *QSnb.cim-5B* can significantly reduce the SNB lesion type by 0.52 and 0.56, respectively, and those with both QTL always displayed the lowest lesion type (Fig. 4a). When the lines carrying only *QSnb.cim-2A* were compared with those carrying only *QSnb.cim-5B*, the latter group had slightly lower SNB lesion type, although no significant difference was detected (Fig. 4a).

In the KC population, a very similar trend was observed, where lines with a single QTL outperformed those without any of the two QTL, and those having both QTL demonstrated the lowest SNB lesion type (Fig. 4b). As for the lines with *QSnb.cim-2A* and those with *QSnb.cim-4B*, the former group had a slightly lower lesion type (Fig. 4b).

### Validation of *QSnb.cim-2A* in the CC population

**Fig. 5:**
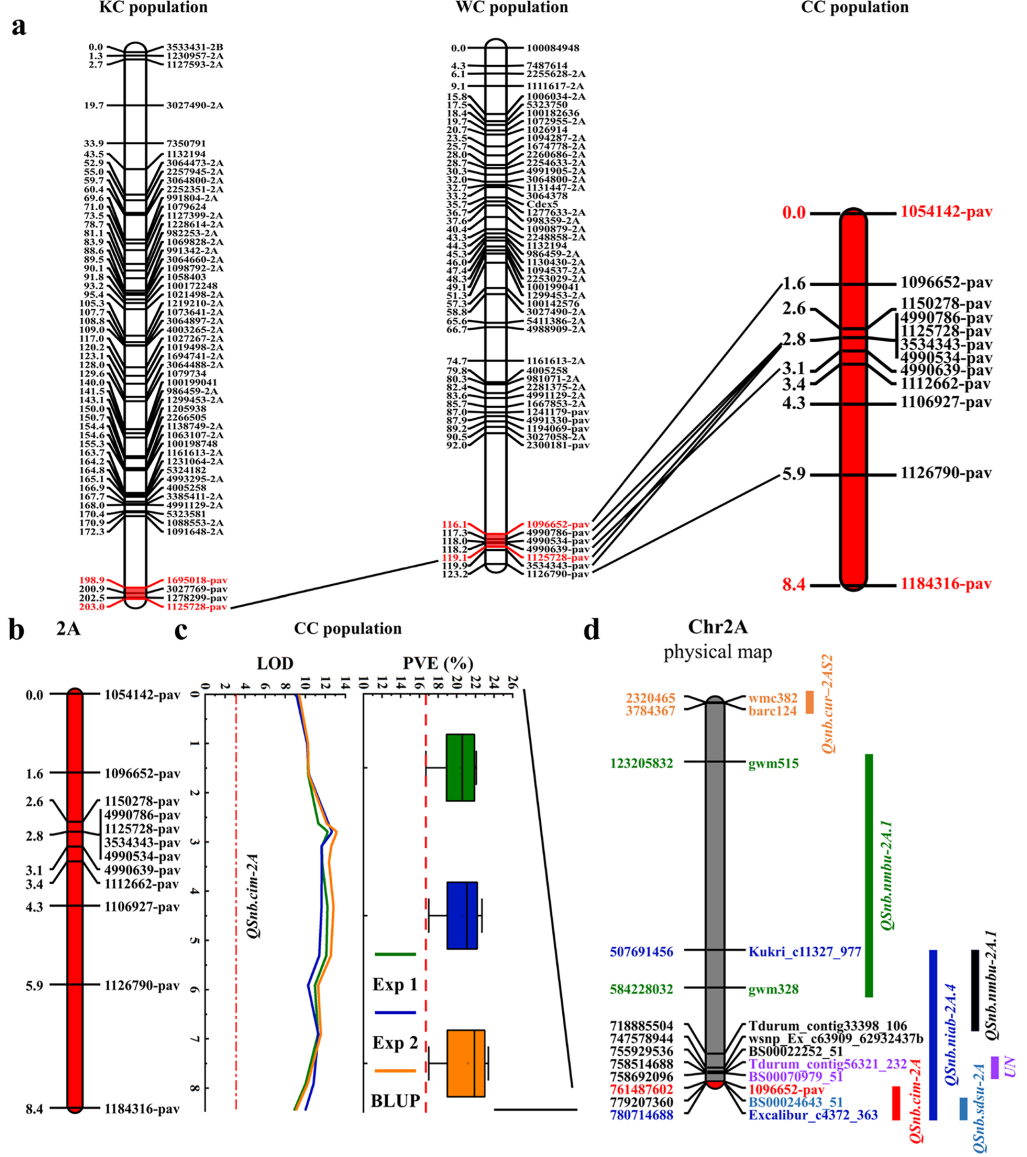
Analysis of *QSnb.cim-2A*. **a** Collinearity of the QTL across the three populations. Only framework markers are shown in the linkage maps. Linkage group segments highlighted in red denote the ranges of *QSnb.cim-2A* in different populations, with the flanking markers delimiting the QTL also shown in red. **b** A new genetic map for the distal region of chromosome 2AL in the CC population. **c** Logarithm of odds (LOD) and phenotypic variation explained (PVE) for *QSnb.cim-2A* in the CC population. **d** Comparison of *QSnb.cim-2A* with previously reported QTL on chromosome 2A

The chromosome region corresponding to *QSnb.cim-2A* was not covered by a previous version of the 2A linkage group in the CC population. Therefore, 12 PAV markers on the chromosome region were added to this population, forming a short linkage group independent of the existing 2A group (Fig. 5a, b). QTL mapping revealed a significant QTL on this short linkage group, accounting for phenotypic variations from 16.70% to 23.40% (Fig. 5c).

### Stacking of *QSnb.cim-2A*, *QSnb.cim-4B*, and *QSnb.cim-5B* in the CC population

**Fig. 6:**
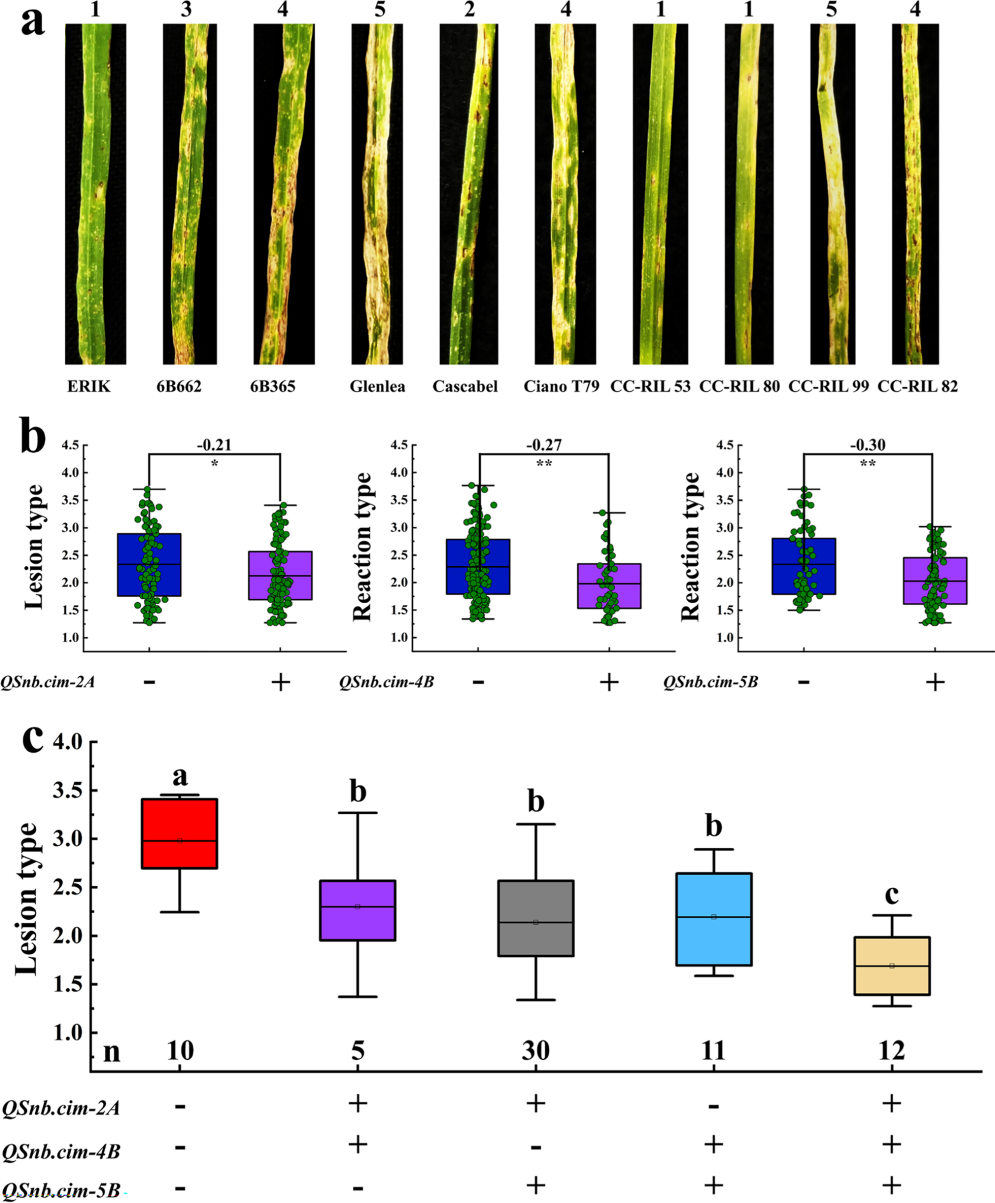
Validation of *QSnb.cim-2A*, *QSnb.cim-4B*, and *QSnb.cim-5B* in the CC population. **a** Phenotypic variation among the parents and selected RILs. The numbers 1 to 5 represent the different SNB lesion types. ERIK, 6B662, 6B365, and Glenlea were used as checks for lesion types 1, 3, 4, and 5, respectively. **b** Effects of *QSnb.cim-2A*, *QSnb.cim-4B*, and *QSnb.cim-5B* for SNB resistance, respectively, and differences between the compared groups are labeled at the top of the box plots. ** and * represent significance at *P* < 0.01 and *P* < 0.05, respectively. **c** Phenotypic effects of different allelic combinations of *QSnb.cim-2A*, *QSnb.cim-4B*, and *QSnb.cim-5B*. + and − represent RILs with and without the target QTL, respectively, and n stands for the number of lines

A wide range of SNB resistance was observed in the CC populations (Fig. 6a). All three QTL, *QSnb.cim-2A*, *QSnb.cim-4B*, and *QSnb.cim-5B*, were verified in this population and were associated with significant reduction in the SNB lesion type by 0.21, 0.27, and 0.30, respectively (Fig. 6b). It is worth noting that *Rht-B1b* and *tsn1* also significantly reduced the SNB lesion type by 0.32 and 0.28, respectively (Additional file 2: Figure S1). Compared with those without any of the QTL, RILs with two QTL exhibited a significant reduction in SNB, and those with all three QTL displayed the lowest infection. No significant differences were identified among the groups having two of the three QTL (Fig. 6c).

## Discussion

It is important to identify resistant plant materials and further mine resistance genes to evaluate their effects in phytopathology studies (McDowell and Woffenden 2003). In the current study, *QSnb.cim-2A* was the only common QTL with major effects identified in all three populations. Since the QTL overlaps with *QSnb.niab-2A.4* (Lin et al. 2020) and *QSnb.sdsu-2A* (AlTameemi et al. 2021) in its physical location, it is likely that these loci are the same QTL (Fig. 5d). Although this QTL has been previously reported using the GWAS method, the current study identified it through bi-parental populations, and its PVE values were higher than those reported in other studies (Downie et al. 2018; Phan et al. 2021; Peters Haugrud et al. 2023). It's worth noting that *QSnb.cim-2A* (or other QTL in the region) has been identified in materials from different countries/regions such as Europe, the USA, Australia, and Mexico (CIMMYT), indicating that this QTL may have a good potential for SNB resistance breeding (Downie et al. 2018; AlTameemi et al. 2021; Phan et al. 2021).

However, *QSnb.cim-2A* was not reported by Hu et al. (2019), in which the same population was utilized for mapping SNB resistance. The reason was that the QTL region was not covered by SNP markers in that study, and it was detected only when PAV markers were added in the current study, although the newly constructed small linkage group (LG) was not linked to the main 2A LG in the CC population. Therefore, when constructing a genetic map, it is essential to pay attention to markers that are not linked to the constructed LGs. Additionally, attention should be paid to chromosomal regions not covered by the available markers, as they may harbor undetected QTL (Slate 2005). This ensures the completeness of the genetic map, thereby facilitating more reliable identification and analysis of QTL.

As mentioned before, *QSnb.cim-2A* was consistently detected across all three populations, each of which shared the same susceptible parent, CIANO T79. Given that the resistant parents (WUYA, KATH, and CASCABEL) are genetically distinct, the consistent presence of this QTL suggests that it is more likely associated with a susceptibility factor derived from CIANO T79, rather than a common resistance allele present in all three resistant parents. This interpretation is consistent with the well-established model of wheat-*P. nodorum* interactions, in which NEs produced by the pathogen are recognized by host sensitivity genes, leading to increased disease susceptibility in an inverse gene-for-gene manner (Faris and Friesen 2020). It is therefore plausible that CIANO T79 carries an uncharacterized NE sensitivity gene responsible for the observed SNB susceptibility, and that *QSnb.cim-2A* may reflect the location of this gene. Although our results support this hypothesis, further validation is necessary to confirm the underlying mechanism. Therefore, breeders should consider that eliminating susceptibility factors, rather than introgressing resistance genes, might be a more effective strategy for enhancing SNB resistance. Nevertheless, we recognize that alternative explanations cannot be completely ruled out, and additional research is required to clarify the genetic basis of this QTL.

*QSnb.cim-4B* was mapped to the short arm of chromosome 4B, and it is closely linked to *Rht-B1* (Additional file 2: Figure S1a). A significant positive correlation between SNB and plant height was observed in the KC population (Additional file 2: Figure S1b). This was unexpected because SNB lesion type shows typically a negative correlation with plant height in field experiments (Lu and Lillemo 2014; Francki et al. 2021), due to a disease escape mechanism rather than genetic resistance. The same mechanism was also found in other diseases at the adult-plant stages in field experiments, such as Fusarium head blight, Septoria tritici blotch, and spot blotch (Lu et al. 2013; Juliana et al. 2022; Patial et al. 2024). However, the current study was conducted at the seedling stage, when the disease escape mechanism should not be present, let alone the fact that the observed positive correlation contradicts this mechanism. This strongly suggests that *Rht-B1* should not be the underlying gene for *QSnb.cim-4B*. Unfortunately, the QTL region delimited in this study is too big to conduct a reasonable candidate gene analysis, and future efforts are needed to identify the underlying gene for the QTL.

Regarding *QSnb.cim-5B*, its underlying gene is likely *tsn1*, which has been documented in numerous previous studies (Liu et al. 2004; Hu et al. 2019). This was based on the result that *tsn1* shared a similar physical location with *QSnb.cim-5B* and the lines harboring *tsn1* exhibited a significant reduction in the SNB lesion type by 0.28 (Additional file 2: Figure S1)*.* This QTL displayed significant effects in the WC and CC populations, but not in the KC population, which was in agreement with the fact that both WUYA and CASCABEL carry the *tsn1* allele, whereas KATH and CIANO T79 possess the *Tsn1* allele (data not shown). Similar to the strategy suggested for *QSnb.cim-2A*, these findings indicate that effective breeding for SNB resistance should focus on eliminating the *Tsn1* susceptibility allele from breeding lines, rather than introgressing *tsn1* from resistant sources.

Based on these results, the combination of the three identified QTL significantly reduced the SNB lesion type and plant height, providing valuable genetic resources for wheat breeding programs. Notably, although no major and stable QTL were identified in CIANO T79, several minor QTL were detected, such as those on chromosomes 1B and 6D (Hu et al. 2019). These minor QTL, together with the major QTL, may have additive effects and collectively contribute to enhanced SNB resistance. Given their consistent phenotypic effects, these QTL, particularly *QSnb.cim-2A* and *QSnb.cim-5B*, could be incorporated into marker-assisted selection strategies. In addition to introgressing resistance-associated alleles, breeding efforts should also focus on eliminating susceptibility alleles such as *Tsn1*, which may play a key role in determining SNB susceptibility in certain genetic backgrounds.

## Conclusions

In this study, two RIL populations were evaluated for SNB resistance at the seedling stage under greenhouse conditions. Using the previously reported genetic map, additional PAV markers were added to create genetic maps with higher marker density, enabling the identification of three major and stable QTL associated with SNB resistance. Specifically, the QTL designated as *QSnb.cim-2A* was identified in both the WC and KC populations and was subsequently validated in the CC population. The other two QTL, *QSnb.cim-4B* and *QSnb.cim-5B*, were found to be associated with *Rht-B1b* and *tsn1*, respectively. The combined effect of these three QTL was found to significantly reduce susceptibility to SNB. Therefore, incorporating these QTL into wheat breeding holds great promise for enhancing SNB resistance.

## Materials and methods

### Plant materials

Two elite CIMMYT breeding lines, WUYA (WAXWING*2/CIRCUS) and KATH (WHEAR/KRONSTAD F2004), have shown good resistance to spot blotch and SNB in our previous studies (Singh et al. 2018; Hu et al. 2019; He et al. 2020). In this study, the bi-parental populations developed from crosses of these two resistant lines with the common susceptible cultivar CIANO T79 (BUCKY/(SIB)MAYA-74/4/BLUEBIRD//HD-832.5.5/OLESEN/3/CIANO-67/PENJAMO-62) were used. The two populations, hereafter referred to as WC (WUYA/CIANO T79) and KC (KATH/CIANO T79), were developed via the single-seed descent method, and each population consisted of 232 F_2:7_ RIL progenies (Singh et al. 2018; He et al. 2020). Additionally, a third bi-parental population was used for validation of the major and stable QTL identified in the current study. It was developed through hybridizing CASCABEL (SOKOLL//W15.92/WBLL1) with CIANO T79 (referred to as the CC population), including 226 F_2:7_ lines (Hu et al. 2019). In this study, Erik, 6B662, 6B365, and Glenlea were used as checks for resistance (R), moderate resistance (MR), moderate susceptibility (MS), and susceptibility (S), respectively, based on their well-documented and consistent responses to SNB worldwide.

### Disease screening protocols

*Parastagonospora nodorum* isolate MexSn4, collected from Tlalnepantla (Mexico) in September 2007, was used in this study. The cultures were grown on modified V8 agar medium (7.5 mL 30% V8-juice, 0.5 g agar, 0.1 g calcium carbonate, and 17.5 mL distilled water) in Petri dishes of 10-cm diameter at room temperature (Liu et al. 2004; Singh et al. 2007; Hu et al. 2019). Fresh *P. nodorum* cultures were prepared by rubbing a 1-cm diameter plug of stock cultures onto a new, fresh V8 medium, and pink pycnidia were visible on the surface of the V8 medium after a week (Singh et al. 2007). A spore suspension was prepared by flooding each Petri dish with sterile distilled water and gently brushing the V8 medium surface to liberate the spores. The resulting suspension was then filtered through four layers of cheesecloth. Subsequently, the spore concentration was adjusted to 1.0** × **10^7^ spores/mL, and Tween 20 (two drops per 100 mL of spore inoculum) was added before inoculation (Singh et al. 2007).

The three populations (WC, KC, and CC) were planted in a greenhouse with day and night temperatures of 22°C and 18°C, respectively, to evaluate SNB response at the seedling stage. A randomized complete block design was used for each of the two experiments, with two replicates per experiment. For each entry, four plants were grown in plastic containers as an experimental unit. The four plants within each unit were individually assessed, and their values were averaged to obtain the mean for subsequent analysis (Hu et al. 2019). The seedlings were inoculated at 14 days after sowing, when the second leaf was fully expanded. A hand sprayer was used to apply the inoculum to the plants until runoff occurred. After the leaves had dried, the plants were placed in a misting chamber for 24 h to promote infection and were then returned to the greenhouse (Hu et al. 2019). A scale of 1 to 5 was used to evaluate SNB infection types at 7 days post-inoculation, with 1 indicating high resistance and 5 indicating high susceptibility (Eyal 1999; Feng et al. 2004).

### Genetic map construction and QTL analysis

The three populations have been genotyped in previous studies using the DArTseq genotyping-by-sequencing (GBS) platform at the Genetic Analysis Service for Agriculture at CIMMYT, Mexico (Singh et al. 2018; Hu et al. 2019; He et al. 2020). The available genetic maps were constructed using SNP markers, and some presence/absence (PAV) markers from the DArTseq GBS platform were added in the current study to fill genetic gaps in the critical QTL regions, as determined by JoinMap 4.0 software. Chromosomal locations of the PAV markers were determined via BLAST searches against the Chinese Spring reference genome and linkage analysis (Additional file 1: Table S1). Linkage maps were drawn with MapChart 2.2 (http://www.biometris.nl/uk/Software/MapChart/) (Voorrips 2002).

The WC and KC populations were used to identify SNB resistance QTL using the MapQTL 6.0 software package (https://www.kyazma.nl/index.php/MapQTL/) with a ‘multiple QTL model mapping’ (MQM) algorithm (Van Ooijen 2009). A LOD threshold of 3.10 was set for declaring significant QTL based on 1000-permutation tests. The multi-environment traits (MET) algorithm of IciMapping 4.1 (LOD score values ≥ 5.00) was used to confirm the stable and major QTL identified by MQM (Meng et al. 2015). QTL names were assigned according to International Rules of Genetic Nomenclature (http://wheat.pw.usda.gov/ggpages/wgc/98/Intro.htm), with ‘Snb’ and ‘cim’ standing for ‘Septoria nodorum blotch’ and ‘CIMMYT’, respectively.

### Statistical analysis

The lme4 package for R software was used to calculate the Best linear unbiased prediction (BLUP) values (Bates et al. 2015). The PROC GLM module of SAS V8.0 software (SAS Institute, Cary, NC, USA; https://www.sas.com) was performed for the analysis of variance (ANOVA). IBM SPSS Statistic 26 (SPSS, Chicago, IL, USA; https://en.wikipedia.org/wiki/SPSS) was used to perform Student’s *t*-test (*P* < 0.05) and correlation analysis. Origin 2021 software (https://www.originlab.com/) was used to draw box plots.

## Supplementary Information

**Additional file 1: Table S1.** Physical location information for the important PAV markers on chromosome 2A. **Table S2.** QTL detected in the QTL × environment (QE) interaction module for SNB in the WC and KC populations.

**Additional file 2: Figure S1.** Analysis of *QSnb.cim-4B* and *QSnb.cim-5B*.

## Data Availability

The datasets used and/or analyzed during the current study are available from the corresponding author upon reasonable request.

## Abbreviations

CC: CASCABEL/CIANO T79 population
KC: KATH/CIANO T79 population
NEs: Necrotrophic effectors
PAV: Presence/absence variation
QTL: Quantitative trait loci
RIL: Recombinant inbred lines
SNB: Septoria nodorum blotch
WC: WUYA/CIANO T79 population
