# Supplementary material for: Identification and validation of a major QTL on chromosome 2A for wheat-Parastagonospora nodorum interactions
Source: Phytopathol Res. 2025 Sep 24;7(1):82. doi: 10.1186/s42483-025-00371-z (PMC12457223; doi:10.1186/s42483-025-00371-z)
Supplement: Supplementary file 2 — Additional file 2: Figure S1. Analysis of QSnb.cim-4B and QSnb.cim-5B. [file 42483_2025_371_MOESM2_ESM.docx]

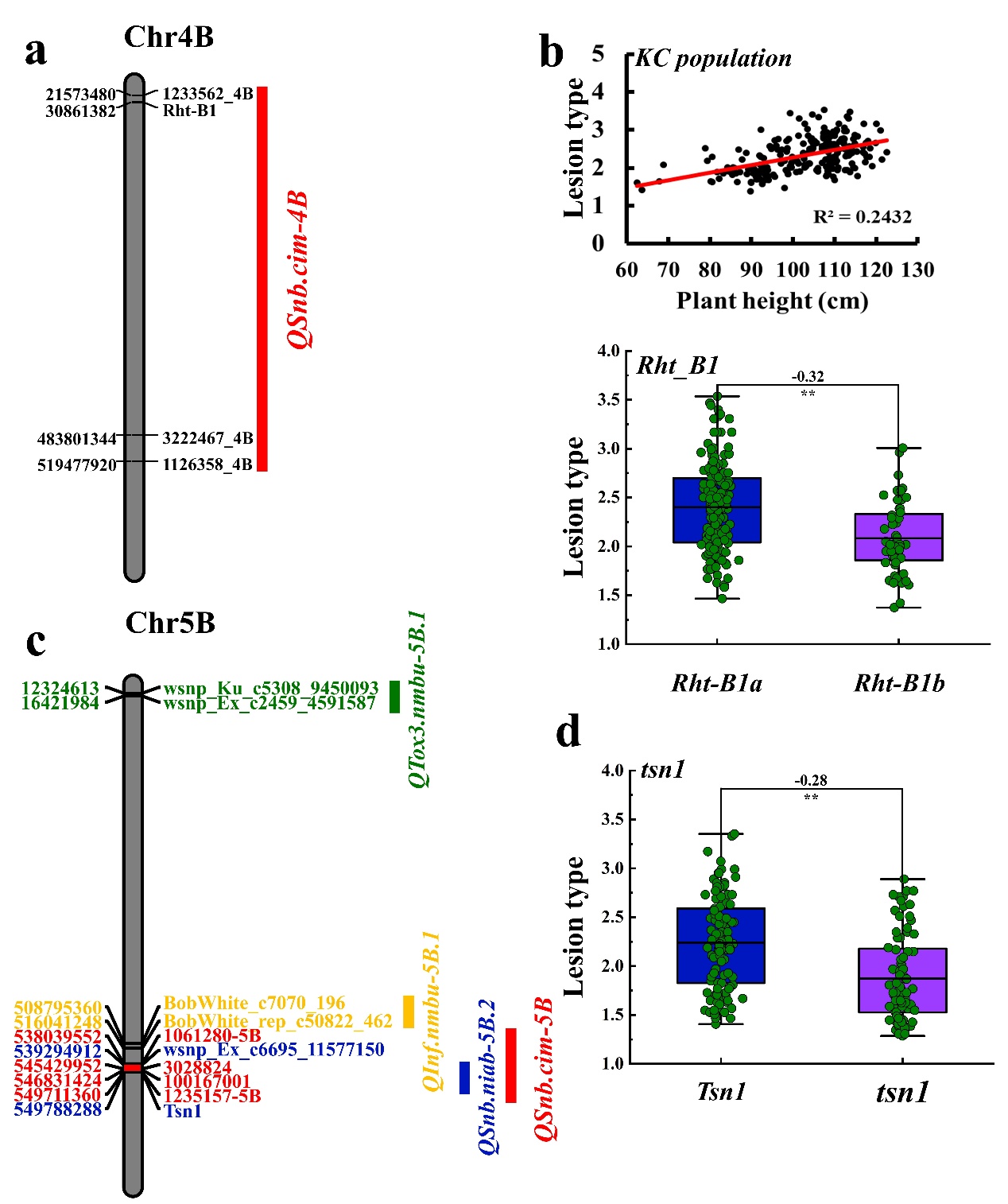


**Figure S1** Analysis of *QSnb.cim-4B* and *QSnb.cim-5B*. **a** The QTL region of *QSnb.cim-4B* harboring *Rht-B1*. **b** Regression analysis of SNB and plant height (top) and the effect of *Rht-B1* on SNB (bottom). **c** Comparison of *QSnb.cim-5B* (delimited by markers in red) with previously reported QTL on chromosome 5B. **d** The effect of *tsn1* on SNB.
